# Synergistic effect of Indium and Gallium co-doping on growth behavior and physical properties of hydrothermally grown ZnO nanorods

**DOI:** 10.1038/srep41992

**Published:** 2017-02-03

**Authors:** Jun Hyung Lim, Seung Muk Lee, Hyun-Suk Kim, Hyun You Kim, Jozeph Park, Seung-Boo Jung, Geun Chul Park, Jungho Kim, Jinho Joo

**Affiliations:** 1 School of Advanced Materials Science and Engineering, Sungkyunkwan University, Suwon, Gyeonggi 440-746, Korea; 2 Department of Nanomaterials Engineering, Chungnam National University, Daejeon 305-764, Korea; 3 Department of Materials Science and Engineering, KAIST, Daejeon 305-338, Korea; 4 Institute for Superconducting and Electronic Materials, University of Wollongong, North Wollongong, NSW 2500, Australia

## Abstract

We synthesized ZnO nanorods (NRs) using simple hydrothermal method, with the simultaneous incorporation of gallium (Ga) and indium (In), in addition, investigated the co-doping effect on the morphology, microstructure, electronic structure, and electrical/optical properties. The growth behavior of the doped NRs was affected by the nuclei density and polarity of the (001) plane. The c-axis parameter of the co-doped NRs was similar to that of undoped NRs due to the compensated lattice distortion caused by the presence of dopants that are both larger (In^3+^) and smaller (Ga^3+^) than the host Zn^2+^ cations. Red shifts in the ultraviolet emission peaks were observed in all doped NRs, owing to the combined effects of NR size, band gap renormalization, and the presence of stacking faults created by the dopant-induced lattice distortions. In addition, the NR/p-GaN diodes using co-doped NRs exhibited superior electrical conductivity compared to the other specimens due to the increase in the charge carrier density of NRs and the relatively large effective contact area of (001) planes. The simultaneous doping of In and Ga is therefore anticipated to provide a broader range of optical, physical, and electrical properties of ZnO NRs for a variety of opto-electronic applications.

The unique physical and optical properties of zinc oxide (ZnO), such as the wide band gap of 3.37 eV and the large exciton binding energy of 60 meV, make this material attractive for a broad range of optoelectronic applications^1^,^2^,^3^,^4^,^5^,^6^,^7^,^8^. Considerable research on ZnO has thus been carried out owing to its potential use in ultraviolet light emitting diodes (LEDs), lasers, or sensors, usually in the form of p/n junction devices.

Although it has conventionally been reported that homojunction devices are more efficient than heterojunction devices in terms of band offset and/or energy barrier engineering, homojunction devices based on ZnO exhibit relatively poor electrical characteristics compared to their heterojunction counterparts due to the p-type doping difficulty in ZnO. Therefore, in order to form p/n junctions, most of the reported ZnO-based diodes and LEDs to date involve the use of p-type materials such as GaN, polymers, Si, graphene, CuAlO_2_, and CuSCN^9^,^10^,^11^,^12^,^13^. In such devices, the charge transport efficiency is relatively low because of the large band offsets and mechanical strains induced at the junction interface. Such a disadvantage can be overcome by applying nano-structured junctions that increase the effective contact areas. In this regard, one-dimensional nanostructures are intensively studied in order to enhance the carrier injection efficiency and their recombination rates^14^,^15^,^16^,^17^.

Another approach for the enhancement of the optoelectronic properties of ZnO is to dope ZnO with group III, IV, and V elements such as Al, Ga, In, Sn, and Sb^18^,^19^,^20^,^21^,^22^. Incorporation of doping elements in ZnO nanorods (NRs) or nanowires have been synthesized by a number of research groups. In-doping was shown to be effective at increasing their electrical conductivity without compromising the optical transparency^20^,^21^. Ga is also a relatively stable doping element, which is much less likely to induce undesired chemical reactions than other impurities^17^,^21^,^22^.

In previous our reports, In or Ga-doped ZnO NRs were synthesized using simple and cost effective hydrothermal methods, allowing relatively easy incorporation of doping elements in ZnO even at low temperatures^23^,^24^,^25^,^26^. It was found that In or Ga doping influences the microstructure of the NRs. Also, the optical and electrical properties of heterojunction devices were closely related to the charge carrier density and defect states within the NRs to a large extent. The electrical conductivity of the NRs was shown to increase with increasing doping concentration to a certain extent. Excessive doping of ZnO NRs with either In^3+^ or Ga^3+^ cations that are larger or smaller than Zn^2+^ cations, respectively, may induce significant mechanical stress and strain in the host matrix. This phenomenon generates undesired microstructural defects that deteriorate the optoelectronic properties of ZnO NRs. It can thus be anticipated that the co-doping of ZnO NRs with both Ga and In dopants will increase their electrical conductivity by providing high carrier densities, while preserving a relatively stress-free microstructure. This is because the presence of cations that are both larger (In^3+^) and smaller (Ga^3+^) than the host Zn^2+^ cations is highly likely to release the net mechanical stress/strain that would be induced with only a single dopant.

To the best of our knowledge, the effects of In and Ga co-doping in ZnO NRs using hydrothermal methods have not been reported in the literature to date. Therefore, in the present study, the hydrothermal synthesis of In- and Ga-doped ZnO NRs is highlighted, especially focusing on In + Ga co-doped ZnO NR arrays on the surface of p-type silicon and GaN substrates. The effects of single doping and co-doping on the microstructure, crystal defects, and optoelectronic properties of ZnO NRs are examined.

## Results

**Figure 1:**
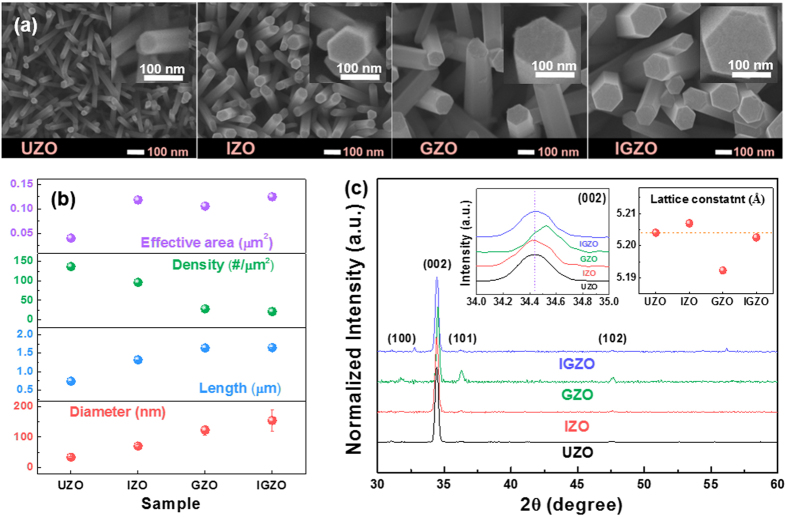
(**a**) Top-view FE-SEM images of the UZO, IZO, GZO, and IGZO NRs. (**b**) Average effective area of the (001) plane, NR areal density, NR diameter and NR length of the UZO, IZO, GZO, and IGZO NRs. (**c**) XRD patterns of the NRs. Insets show the enlarged (002) peaks and the average c-lattice parameters calculated from the 2θ values of the (002) peaks.

To investigate the effect of In, Ga, and In + Ga doping on the morphology and size of the ZnO NRs, top-view FE-SEM images were collected as shown in Fig. 1a. The undoped ZnO and In, Ga, and In + Ga doped ZnO NRs are denoted as UZO, IZO, GZO, and IGZO, respectively, hereafter. All samples clearly exhibit rod shapes with flat end top facets. The average diameter and length, the number of the NRs per unit area (density), and the effective surface area of the (001) planes, were estimated from the SEM images and are plotted in Fig. 1b (cross-sectional images not shown here). The corresponding values for the UZO NRs are approximately 34.2 ± 1.99 nm, 0.74 ± 0.02 μm, 137 rods/μm^2^, and 0.04 μm^2^, respectively. Upon doping, the average diameter, length, and effective area increased significantly relative to the undoped ZnO NRs. However, the number of NRs per unit area decreased. Especially, the average diameters of the IZO, GZO, and IGZO NRs are approximately 2-, 4-, and 5-times larger than that of UZO, respectively. The results indicate that the lateral growth of the NRs is stimulated by the incorporation of Ga to a larger extent than with In. Further increase in lateral growth is observed when both Ga and In are present, which implies that a synergetic effect of In + Ga co-doping can exist.

The growth reactions of the doped ZnO NRs are proposed as follows^27^,^28^,^29^,^30^:


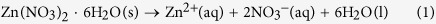



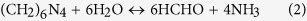



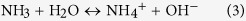



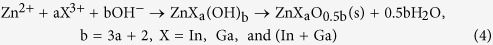


It is well known that each polar (001) plane consists of either Zn or O atoms, while non-polar (110) and (100) planes contain an equal number of both elements^31^. The metastable polar surface stimulates the columnar growth owing to its relatively high surface energy (2.0 J/m^2^ for {001} planes) compared to the 6-fold vertical planes (1.16 J/m^2^ for {100} planes)^32^,^33^. In addition, the adhesion of NH_4_^+^ ions from hexamethylenetetramine (HMT) in Eq. (3) on the sidewalls suppresses lateral growth by inhibiting the adsorption of Zn^2+^ ions on the sidewalls. Therefore, faster growth along the vertical c-axis occurs in NR structures^34^.

The significant increase in diameter of the doped NRs can be interpreted as the result of two mechanisms^25^,^26^,^35^,^36^,^37^; (1) the inhibition of heterogeneous ZnO nucleation and (2) the reduced concentration of OH^−^ ions in the solution. As described in our previous studies^25^,^26^, the incorporation of dopants in ZnO could increase the energy barrier for the formation of a crystal nucleus according to the classical nucleation model. In addition, the In or Ga ions decreased concentration of oxygen precursor because they possibly create complexes with OH^−^ in the solution. Both the increased energy barrier and the decreased reaction kinetics between Zn^2+^ and OH^−^ reduced the heterogeneous nucleation of the ZnO. The low density of ZnO nuclei promotes the lateral growth, resulting in the larger diameters of the doped ZnO NRs. The above two mechanisms might also account for the larger diameter of the IGZO NRs than IZO and GZO NRs in this work. The total dopant concentration of the initial IGZO solution (0.005 M) is higher than that of the IZO and GZO solutions (0.0025 M): the lateral growth of IGZO NRs is thus expected to occur faster. In addition, the increased length of the doped NRs is likely to result from the more positively charged polar surfaces due to the incorporation of In^3+^ or Ga^3+^ ions as shown in Fig. S1. When the trivalent dopants substitute the divalent Zn sites in the Zn-terminated surface, the incoming oxygen ions can adsorb more readily on the growing surface, resulting in enhanced vertical growth rates (Fig. S1). It is also noted that the average length of the GZO NRs is somewhat greater than that of the IZO NRs. This is partly attributed to the different polarities of the (001) planes, resulting from the higher electronegativity of Ga (χ = 1.81) than that of In (χ = 1.78).

Figure 1c shows the X-ray diffraction (XRD) patterns of the UZO, GZO, IZO, and IGZO NRs. All peaks match well with the positions reported for wurtzite ZnO [ICDD No. 36-1451] and no apparent secondary peaks related to the presence of In or Ga are observed. The diffraction patterns indicate that the hexagonal phase of the host matrix is well preserved regardless of the incorporation of Ga or In. The intense (002) peaks in the XRD patterns for all samples indicates that the synthesized NRs have a preferential [001] growth direction. Enlarged (002) peaks are shown as insets in Fig. 1c. With respect to the undoped ZnO (002) peak, the IZO (002) peak shifts towards lower angles while the GZO (002) peak shifts in the opposite direction. The variation in the peak positions is related to the incorporation of the dopants, which influence the c-axis lattice constant by substituting the host Zn^2+^ ions. The c-axis lattice constants were calculated from the (002) peak positions and are also shown as insets in Fig. 1c. The position and full width at half maximum (FWHM) values of the (002) peaks are summarized in Table S1, along with the calculated lattice parameters for all samples. The c-axis lattice parameters of the UZO, IZO, and GZO NRs are approximately 5.204, 5.207, and 5.192 nm, respectively. The variations in the lattice parameters were probably due to the larger radius of In^3+^ ion (~0.81 Å) and smaller radius of Ga^3+^ ion (~0.62 Å) than that of Zn^2+^ ion (~0.74 Å). Considering the relative differences of ionic radius between Zn^2+^ and dopants (In^3+^ and Ga^3+^) and the actual contents of In and Ga in the IGZO NRs (In: 5.04%, Ga: 7.05%, as noted in the experimental section), the c-axis lattice parameter of the IGZO NRs should be slightly smaller than that of the UZO NRs. However, it is noted that the lattice parameter of the IGZO NRs (~5.203 nm) is almost comparable to that of the UZO NRs because of a counterbalance between the lattice expansion and contraction induced by the In and Ga dopants, respectively. This indicates that a lattice strain of the IGZO NRs noticeably decreases as compared to those of the IZO and GZO NRs.

Raman analysis was performed to evaluate the strain variations induced by the dopants. The E_2H_ peak is usually used to analyze the stress state in thin films and nanostructures, due to its high sensitivity to the presence of mechanical stress and/or strain^38^,^39^. With respect to the E_2H_ peaks of the UZO NRs, those of IZO and GZO NRs shift towards opposite directions (Fig. S2), similar to those observed in the XRD (002) peaks (Fig. 1c). The relatively lower (IZO) and higher (GZO) frequencies of the E_2H_ peaks compared to that of the reference (UZO) indicate that elongation and contraction occur along the c-axis, respectively, due to the larger size of the In^3+^ ions and smaller size of the Ga^3+^ ions than that of the host Zn^2+^ ions. The peak frequency of the IGZO NRs is close to that of the UZO, similar to that observed in the XRD results.

**Figure 2:**
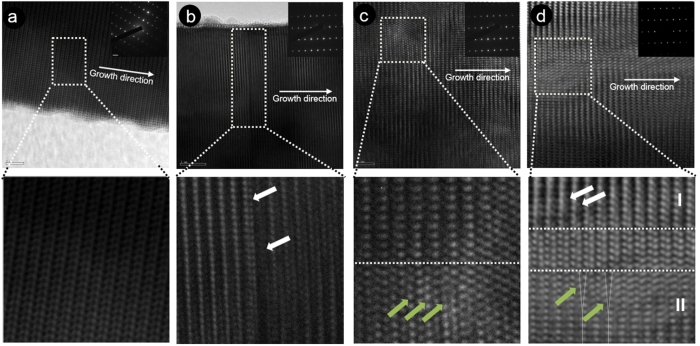
High resolution TEM images of (**a**) UZO, (**b**) IZO, (**c**) GZO, and (**d**) IGZO NRs. The insets in (**a**–**d**) are the corresponding SAD patterns, and four lower pictures are magnified images of the dotted square regions in (**a**–**d**), respectively. The white and green arrows are identified as type-III and type-II intrinsic SFs, respectively.

To obtain visual information on the microstructure of the NRs, high resolution transmission electron microscopy (HRTEM) was performed as shown in Fig. 2. All NRs consist of monocrystalline wurtzite ZnO, as confirmed by the selected area diffraction (SAD) patterns shown as insets in Fig. 2a–d. In the defect-free UZO NRs, the stacking sequence of the Zn cations and O anions in alternating (004) planes is …AaBbAaBbAaBb… as can be seen in Fig. 2a. Capital letters correspond to the metal cations and lowercase letters denote the oxygen anions. Even though the SAED pattern of the doped NRs (IZO, GZO, and IGZO) is a single crystal, we observed that a stacking sequence is interrupted by some apparent irregularity in stacking (indicated by arrows) in Fig. 2b–d.

A magnified image of the dotted square area of Fig. 2(b) shows the presence of the planar defects in IZO NR. White arrows point out the location of the stacking faults (SFs), and the stacking sequence is …AaBbAaBbCcBbAaBbAaBb…, which is known as a type-III intrinsic SF^40^,^41^,^42^. Former reports indicate that planar defects and inversion domain boundaries can form due to the presence of In-O layers in ZnO. Since this type of intrinsic SF does not involve the insertion of external planes in the already existing lattice, the defects observed in the IZO NRs may have formed due to the condensation of oxygen vacancies near the In cations^40^,^41^. As such, the compressive stress caused by the relatively large In cations can be reduced. In the case of GZO NRs, similar defects (indicated by green arrows) are observed in the magnified image of Fig. 2c. The stacking sequence may be described as the sequence of …AaBbAaBbCcAaCcAaC, and the corresponding defect is a type-II intrinsic SF, with the Burgers vector of the partial dislocation being 1/6 < 023 >^40^. The stacking faults in GZO are suspected to be generated by the creation of partial dislocations to release the tensile stress generated by the relatively small Ga cations. The structural defects in doped ZnO NRs are highly likely to form during growth. Interestingly, for the IGZO NRs, both of the above SF types in the IZO and GZO are observed, as shown in the magnified image of Fig. 2d. The SFs of region I and II in Fig. 2d consist of type-III and type-II intrinsic SFs, respectively (indicated by green and white arrows, respectively). From the above observations, the type-III and type-II SFs in the In + Ga co-doped ZnO NRs are likely to be generated by In and Ga cations, respectively.

**Figure 3:**
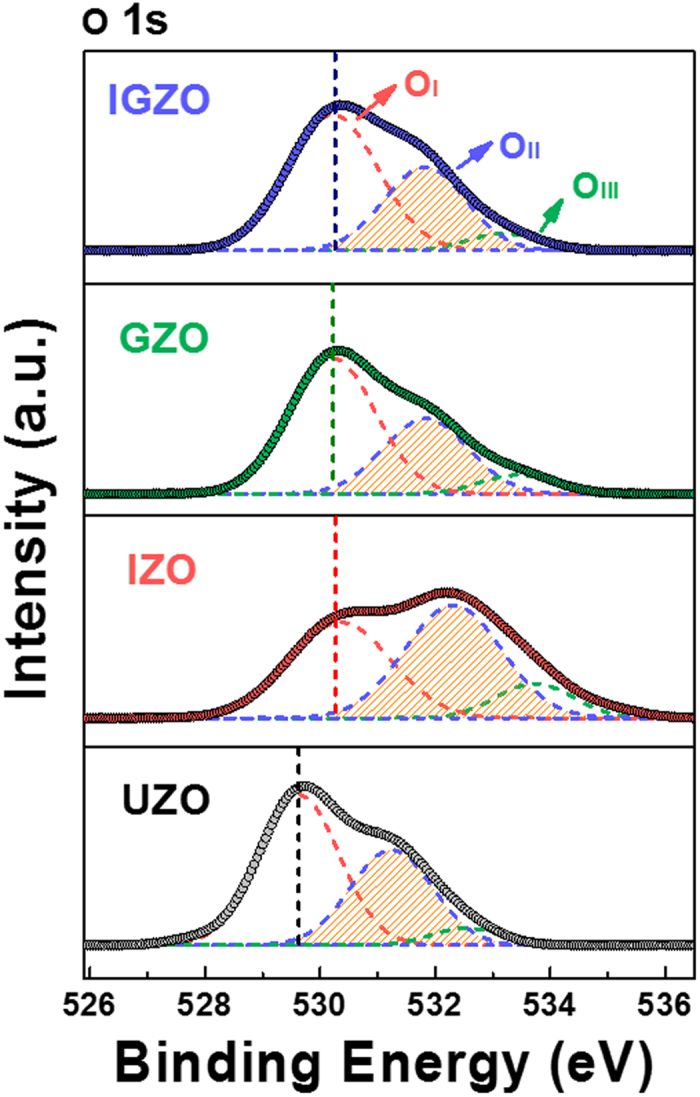
XPS O 1 s spectra of UZO, IZO, GZO, and IGZO NRs. Deconvoluted into 3 distinct sub-peaks, O_I_, O_II_, and O_III_ using Gaussian fitting.

In order to gain insight into the bonding states of the Zn cations and oxygen anions, XPS analyses were performed as shown in Figs 3 and S3. The O 1 s (Fig. 3) and Zn 2p (Fig. S3a) peaks of the doped ZnO NRs shift towards higher binding energies compared to UZO NRs. The shifts in binding energy could be attributed to the formation of stronger bonds between the dopants (In and Ga) and oxygen in doped NRs compared to those between Zn and oxygen in the UZO NRs, owing to higher electronegativity values of In (χ = 1.78) and Ga (χ = 1.81) than that of Zn (χ = 1.65). The In 3d and Ga 2p peaks are clearly seen from the IZO and GZO samples, respectively (Fig. S3b). Both In 3d and Ga 2p peaks are also observed in the XPS spectrum collected from the IGZO NRs. The above results indicate that In, Ga, and In + Ga have indeed blended into the ZnO matrix to form IZO, GZO, and IGZO NRs, respectively.

In addition, the asymetric peaks observed in the O 1 s spectra (Fig. 3) can be deconvoluted into 3 distinct sub-peaks (O_I_, O_II_, and O_III_) using Gaussian fitting. The positions and relative integrated peak intensity ratios of the O 1 s sub-peaks from all samples are listed in Table S2. Among them, the low energy O_I_ peak originates from the O^2−^ ions forming bonds with the metal cations (Zn^2+^, Ga^3+^, and In^3+^) in the lattice. The medium energy O_II_ component is associated with the O^2−^ ions near the oxygen deficient regions (where oxygen vacancies are present) within the ZnO matrix, which may be considered as a parameter describing the relative amounts of oxygen vacancies between the samples^43^. The O_II_/O_total_ intensity ratios for the UZO, IZO, GZO, and IGZO NRs are calculated to be 37.2%, 46.8%, 32.8%, and 33.6%, respectively. Notably, the IZO samples exhibit a higher O_II_ /O_total_ ratio than the reference UZO, indicating that In doping weakens the average metal-oxygen bond energy, thereby inducing more oxygen vacancies in the ZnO lattice. This result is acceptable because the In-O bonding energy is lower than that of Zn-O^44^. In contrast, the O 1 s peak of the GZO exhibits a somewhat different configuration (the positions of the deconvoluted sub-peaks and their relative intensities): the peak ratio related to the metal-oxygen bonds (O_I_/O_total_) increases and the oxygen deficiency peak ratio (O_II_/O_total_) decreases in comparison with those of UZO NRs. This may be attributed to the stronger Ga-O bond energy than those of Zn-O and In-O^45^,^46^. For the IGZO NRs, the O 1 s peak configuration is similar to that of the GZO NRs but the O_I_/O_total_ and O_II_/O_total_ intensity ratios increase slightly, up to 60.5% and 33.6% compared to 59.9% and 32.7% for the GZO NRs, respectively. This indicates that stronger metal-oxygen bonds are formed in the IGZO NRs than in the GZO NRs, while the concentration of oxygen vacancies does not decrease. The strong metal-oxygen bonds are attributed to the presence of Ga dopants, and the slight increase in oxygen vacancies could be due to the presence of In dopants that form relatively weak In-O bonds.

**Figure 4:**
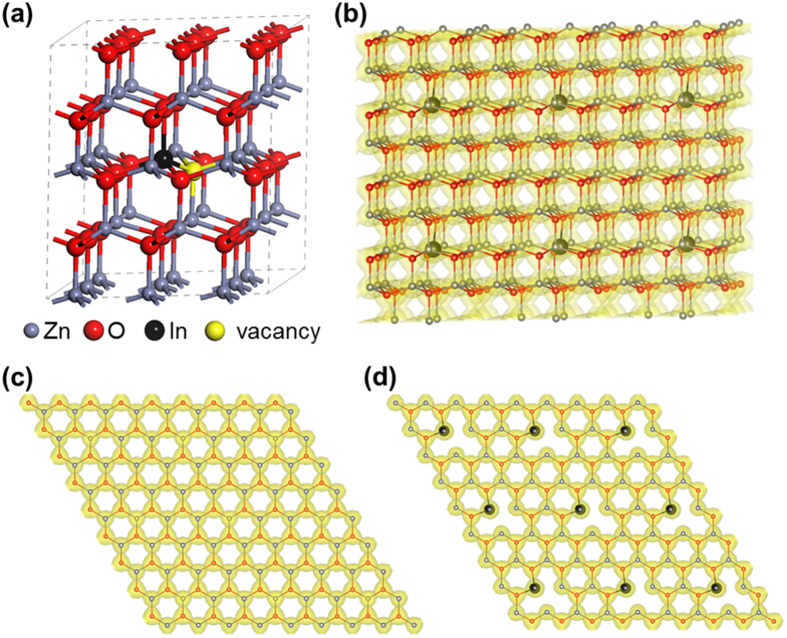
(**a**) Morphology of the IZO (or GZO) super cell. (**a**) Atomic geometry of the IZO super cell. (**b**) Spatial electron density over the IZO supercell. (**c**) In-plane electron density of a defect-free layer composed of Zn and O ions. (**d**) In-plane electron density of an In- and vacancy-rich layer. The GZO supercell was constructed the same way by replacing the In atom with a Ga atom. The yellow area around the atoms represents constant electron density regions (0.06 e/Å^3^).

In order to understand the effects of In and Ga doping on the electronic structure of ZnO NRs, first-principle calculations based on density functional theory (DFT) were performed. A ZnO supercell with 72 atoms is illustrated in Fig. 4a. Here, a Zn atom was replaced with an In atom. This was also carried out to create a GZO supercell, and an IGZO supercell was generated by removing two Zn atoms and replacing each vacant site with a Ga and an In atom.

The formation energy of an oxygen vacancy, *E*_*form*_(*V*_*o*_), can be estimated as follows:


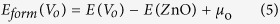


where *E(V*_*o*_) represents the total energy of the ZnO supercell with a single oxygen vacancy, *E(ZnO*) represents the energy of a perfect ZnO supercell, and *μ*_*o*_ represents the chemical potential of oxygen determined by half the energy of an O_2_ molecule. The *E*_*form*_(*V*_*o*_) was calculated by removing the first nearest oxygen ion from the Zn, In, and Ga cations in the UZO, IZO, and GZO supercells, respectively. It was found that the E_*form*_(V_o_) values for the UZO, IZO, and GZO were 3.63, 3.32, and 4.10 eV, respectively. Therefore, the incorporation of In or Ga in ZnO NRs either promotes or suppresses the formation of oxygen vacancies by decreasing or increasing the *E*_*form*_(*V*_*o*_), respectively. The calculations are consistent with the XPS results, regarding the tendency of IZO and GZO NRs to form oxygen vacancies. The energy of electron removal from the supercell, *E*_*removal*_, was calculated in order to understand the effect of the doping element on the density of free electrons as follows:


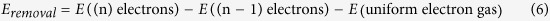


where *E*((n) electrons) and *E*((n−1) electrons) represent the energy of the supercell with the original number of electrons (n), and the energy of the system with (n−1) electrons, respectively. The energy of the uniform electron gas, *E*(uniform electron gas), was used as a reference point. The calculated *E*_*removal*_ of the IZO supercell (−10.2 eV) is lower than that of the GZO supercell (−4.09 eV), showing that IZO has a stronger tendency to release an electron compared to GZO.

The electron charge density of the IZO (or GZO) supercell with oxygen vacancies is illustrated in Fig. 4b–d. The zinc and oxygen ions in the defect-free ZnO supercell form a dense network of electron density (Fig. 4(c)), whereas oxygen vacancies positioned at the first neareast position of In dopants disrupt the local electron density (Fig. 4d). The oxygen vacancies can generate electrons as described in the following equations:


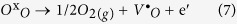



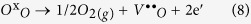


where *O*_2_ is formed from the oxide sublattice 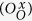 to create charged oxygen vacancies (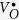 and 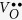) and electrons (e′ and 2e′). It is postulated that although the IZO system is found to be relatively rich in oxygen vacancies (*E*_*form*_(*V*_*o*_)) and free electrons (*E*_*removal*_), the non-continuous electron network can impede the overall charge transport in the system. On the other hand, the Ga dopants with relatively high *E*_*form*_(*V*_*o*_) lower the oxygen vacancy concentration of the system, thus preserving a more continuous electron density network than in IZO. Therefore, In + Ga co-doping is expected to enhance the electron conduction, because both In and Ga dopants release free electrons and reduce oxygen vacancy that acts as a disconnector of the electron network, respectively. The net effect is to induce an increase in free carriers while maintaining a similar order of oxygen vacancy to that of pure ZnO.

**Figure 5:**
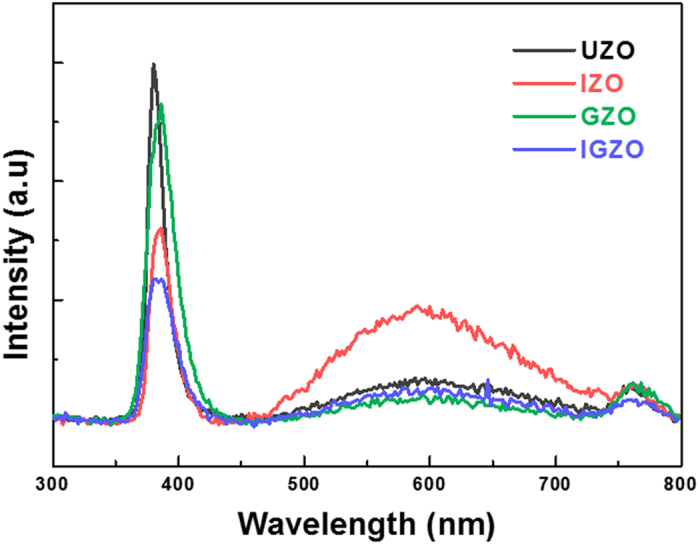
CL spectra of the UZO, IZO, GZO, and IGZO NRs at room temperature.

The cathodoluminescence (CL) spectra of all the NRs were then collected at room temperature in order to evaluate the energy band gap and defect-related visible emissions. As shown in Fig. 5, the UV emission peaks from all doped NRs are red-shifted with respect to undoped NRs. Such a phenomenon is suggested to originate from the following: first, as reported in our previous study, the UV emission peak shifts toward higher wavelengths with increasing In or Ga content, which may be attributed to the increase in NR diameter^25^,^26^,^46^,^47^. This irregular energy shift is caused by the surface resonance effect, in which the surface-to-volume ratio of the NRs becomes smaller with increasing doping levels. The red-shift in the present work may thus be ascribed to the relative increase in NR volume resulting from the incorporation of In, Ga, and In + Ga in the ZnO NRs. Second, the red-shift of the UV peak may be interpreted in terms of band gap renormalization (BGR), which is most likely to originate from the Coulombic interactions between the excess free electrons in the conduction band and electron-impurity scattering^48^. The excess electrons released by the dopants can hinder the transition of free excitons, thus increasing the valence band maximum (VBM) level and reducing the conduction band minimum (CBM). The band gap narrowing (Δ*E*^BGN^) can be expressed by a simple theoretical equation:^49^


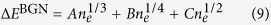


where *n*_*e*_ is the carrier concentration and *A, B*, and *C* are the coefficients of the exchange energy of majority carriers, correlation energy of minority carriers, and carrier-ion interaction energy, respectively. In this equation, the *B* value can be ignored due to low hole concentration in the n-type semiconductors. For the doped samples, the values of *n*_*e*_ and coefficients (*A* and *C*) can increase due to more electrons in the trivalent dopants (In^3+^ and Ga^3+^), and consequently, Δ*E*^BGN^ can increase. As a result, the optical band gap of the doped NRs (*E*_g,doped ZnO NRs_ = *E*_*g*_,_UZO NRs_ −Δ*E*^BGN^) decreases compared to the undoped NRs.

As can be seen from the TEM results (Fig. 2), the SFs induced by the dopants can also affect the band gap energy. It is known that the formation of SFs results in local decreases in band gap because the dopants induce chemical disorder and unsaturated bonds, thus creating localized states in the forbidden energy gap^40^. The distribution of localized states near the band edge depends on the concentration of structural defects in the semiconducting materials, such as oxygen vacancies and SFs. Therefore, a high concentration of localized states in the doped NRs may have contributed to the narrow optical band gap. The red-shift of the UV peak for the doped NRs may thus result from the combined effects of the increased NR size, the BGR effect, and the formation of SFs.

In addition to the UV peak, a broad visible emission band is observed between 458~728 nm in the IZO NRs. The visible emission of the IZO NRs is more pronounced than that of other samples. This relatively high intensity is attributed to the increase in oxygen vacancy concentration, as supported by the previous XPS O 1 s deconvolution results. In contrast, for the GZO and IGZO, the relative intensity decreases, in this case owing to the reduction in oxygen vacancies, despite the incorporation of In ions in the IGZO NRs. As oxygen vacant sites act as radiative centers in the luminescence process, the visible luminescence in ZnO usually originates from the oxygen vacancies. The broad visible emission band is generally considered to occur due to a combination of transitions related to the different charge states of oxygen vacancies (*V*_*O*_, 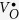, and 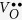)^50^. Consequently, the visible emissions in the CL spectra consist of the superposition of three dominant components near the yellow (2.00 eV, 649 nm), green (2.48 eV, 500 nm), and blue (2.78 eV, 446 nm) regions. The above three types of energy are attributed to the recombination of the 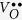 center with delocalized electrons close to the conduction band, the electron transition from 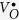 centers to the valence band edge, and the electron transition from neutral oxygen vacancies (*V*_*O*_) to the valence band edge, respectively. The increased visible emission in the IZO NRs is thus suspected to occur due to the transition of electrons from charged oxygen vacancies (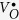 and 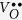) rather than from neutral *V*_*O*_ centers^50^,^51^.

**Figure 6:**
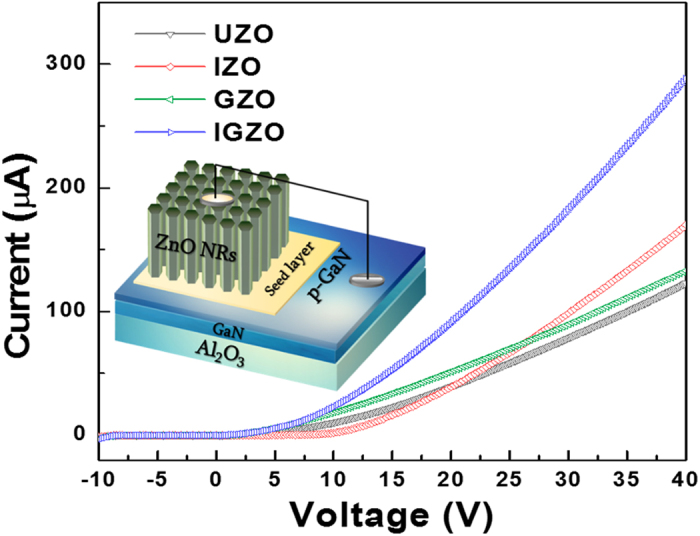
I–V characteristics of the UZO, IZO, GZO, and IGZO/p-GaN heterojunction diodes. A schematic diagram of the device structure is shown as inset.

In order to evaluate the electrical properties of the NRs, heterojunction diodes were fabricated using the UZO, IZO, GZO, and IGZO NRs grown onto p-GaN. The p-GaN acts as a p-type hole injection layer, of which the thickness and hole concentration are approximately 0.36 μm (pre-deposited GaN buffer layer is 2 μm thick) and 7.5 × 10^18^ cm^−3^, respectively. As shown in Fig. 6, the current (*I*) - voltage (*V*) curves for all the samples exhibit diode-like rectification characteristics with a turn-on voltage of approximately 2~3 V. The threshold voltages (*V*_th_), defined as the voltages producing a forward bias current of 1 μA, are approximately 2.57 V, 2.05 V, 2.05 V, and 2.21 V for the UZO, IZO, GZO, and IGZO, respectively. The *V*_th_ values decrease when ZnO NRs are doped, which may be attributed to the band gap narrowing effect as the sub-gap defect states move closer to the bottom of the conduction band^52^,^53^. In addition, the small leakage current under reverse bias may be attributed to not only the contact resistance between the NRs and metal electrode, but also the high defect concentration or trap centers at the NR/p-GaN interface. It is clearly observed that the current increases with increasing applied voltage, but the current values at a given voltage differ: at 40 V, the currents of the UZO, IZO, GZO, and IGZO NRs are 122, 143, 133, and 287 μA, respectively.

Compared to UZO NRs, the current provided by the doped NRs is significantly higher. Furthermore, the current of the IGZO NR diode is considerably higher than that provided by the IZO or GZO NR devices. The superior conductivity of IGZO NRs as determined from its *I-V* slope is suggested to result from the increased effective contact area due to its large cross-sectional diameter and the synergetic effect of In + Ga co-doping on the electron conduction. The latter mechanism involves the generation of abundant free carriers by In and the suppression of oxygen vacancies that disconnect the charge transport network by the addition of Ga. The present report provides valuable insight into enhancing the optoelectronic properties of ZnO NRs by In + Ga co-doping using hydrothermal methods.

## Discussion

In this study, undoped, In-, Ga-, and In + Ga co-doped ZnO NRs were prepared using hydrothermal methods. The effects of In and Ga incorporation on the microstructure, chemical bonding states, and optical properties of ZnO NRs were examined. The average diameter and length of the IGZO NRs increased to a larger extent than those of the IZO and GZO NRs. The average c-axis lattice constant of the IGZO NRs was closest to that of the original ZnO NRs, which is attributed to the compensation of compressive and tensile strains induced by the In^3+^ and Ga^3+^ cations, respectively. From the CL results, the visible emission from the IZO NRs was considerably larger than that of the UZO, GZO, and IGZO NRs, which is interpreted as being due to the relatively high oxygen vacancy concentrations in IZO. The *I-V* characteristics of the diode devices showed that the electrical conductivity of the IGZO NRs was considerably improved, owing to the increased effective contact area and the synergetic effect of In + Ga co-doping. Therefore, it can be concluded that In + Ga co-doping enhances the charge transport capability of the ZnO NRs to a greater extent than with single dopants. IGZO nanorods are thus applicable to a broad range of optoelectronic applications such as short-wavelength semiconductor lasers and light emitting diodes (LEDs).

## Methods

### Experimental

Undoped and In-, Ga-, and In + Ga co-doped ZnO NRs were synthesized on Al_2_O_3_ (001) substrates with an epitaxial GaN (001) layer by low temperature hydrothermal growth. A 0.05 M precursor sol was prepared by dissolving zinc acetate dihydrate (Zn(CH_3_COO)_2_·2H_2_O) in 2-methoxyethanol and then spin-coating on GaN to form ZnO seed layers. After spin coating, the gel films were dried at 150 °C for 10 min to evaporate the solvent and remove organic residues. To grow the ZnO NRs, the seed layer-coated substrate was immersed in DI water containing a mixture of 0.025 M zinc nitrate hexahydrate (Zn(NO_3_)_2_∙6H_2_O) and equivalent molar hexamethylenetetramine (HMT, C_6_H_12_N_4_) at approximately 95 °C for 4 h. To obtain In-, Ga-, and In + Ga co-doped samples, indium nitrate hydrate (In(NO_3_)_3_∙xH_2_O, 0.0025 M), gallium nitrate hydrate (Ga(NO_3_)_3_∙xH_2_O, 0.0025 M), and both precursors (0.0025 + 0.0025 = 0.005 M in total) were added to the previously described aqueous mixture, respectively. The as-grown samples were rinsed with DI water using an ultrasonic cleaner to remove the residual free-standing ZnO particles and then dried at 150 °C on a hot plate in air. The actual contents of In, Ga, and In + Ga for the IZO, GZO, and IGZO NRs were estimated to be 4.45%, 8.10%, and 5.04%/7.05% of the total cation content, respectively, from the integrated areas of the XPS peaks. In order to investigate the pn heterojunction diodes, In electrodes were deposited onto the ZnO NRs and p-GaN films by thermal evaporation.

### Characterization

The morphology and microstructure of the NRs were observed by field emission scanning electron microscopy (FE-SEM, JEOL, JSM7000F) and high-resolution transmission electron microscopy (HRTEM, JEOL, JEM-2100F). The crystallographic information was obtained by X-ray diffraction (XRD, Bruker-AXS, D8 Discover) and Raman spectroscopy (Witec, alpha 300). The chemical bonding structure was analyzed by X-ray photoelectron spectroscopy (XPS, VG Microtech, ECSA2000) using Al Kα radiation, and the binding energies of the XPS spectra were calibrated using the carbon C 1 s peak as reference. The optical properties were measured by cathodoluminescence (CL) spectroscopy (GATAN, MONO CL3 +) at room temperature. In addition, current-voltage (I-V) measurements of the n-ZnO/p-GaN heterojunction diodes were performed with a semiconductor parameter analyzer (Agilent, B1500A).

### Simulation

In order to understand the oxygen vacancy formation energies in the ZnO NRs in the presence of In or Ga dopants, spin-polarized density-functional theory (DFT) calculations in a plane-wave basis were performed using the Vienna ab-initio simulation package (VASP) code and the Perdew-Burke-Ernzerhof generalized gradient approximation (PBE GGA) functional. Valence electrons and core electrons were described by plane waves up to an energy cutoff of 500 eV and the projector augmented wave framework, respectively. A 3 × 3 × 2 wurtzite ZnO supercell with 72 atoms was applied, and 3 × 3 × 3 k-points grid sampling of the Brillouin zones was carried out for all calculations. The final convergence criteria for the electronic wave function and geometry were 10^−4^ eV and 0.02 eV/Å, respectively.

## Additional Information

**How to cite this article**: Lim, J. H. *et al*. Synergistic effect of Indium and Gallium co-doping on growth behavior and physical properties of hydrothermally grown ZnO nanorods. *Sci. Rep.* **7**, 41992; doi: 10.1038/srep41992 (2017).

**Publisher's note:** Springer Nature remains neutral with regard to jurisdictional claims in published maps and institutional affiliations.

## Supplementary Material

Supplementary Information
