# Supplementary material for: Synergistic effect of Indium and Gallium co-doping on growth behavior and physical properties of hydrothermally grown ZnO nanorods
Source: Sci Rep. 2017 Feb 3;7:41992. doi: 10.1038/srep41992 (PMC5290467; doi:10.1038/srep41992)
Supplement: Supplementary Information [file srep41992-s1.pdf]

Synergistic effect of Indium and Gallium co-doping on growth behavior and physical properties of hydrothermally grown ZnO nanorods

Authors: Jun Hyung Lim[1], Seung Muk Lee[1], Hyun-Suk Kim[2], Hyun You Kim[2], Jozeph Park[3], Seung-Boo Jung[1], Geun Chul Park[1,*], Jungho Kim[4,*] & Jinho Joo[1,*]

Correspondence and requests for materials should be addressed to J. J. (jinho@skku.edu) or G. C. P. (pgc1117@skku.edu) or J. K. (jhk@uow.edu.au)

[1]School of Advanced Materials Science and Engineering, Sungkyunkwan University, Suwon, Gyeonggi 440-746, Korea.

[2]Department of Nanomaterials Engineering, Chungnam National University, Daejeon 305-764, Korea.

[3]Department of Materials Science and Engineering, KAIST, Daejeon 305-338, Korea.

[4]Institute for Superconducting and Electronic Materials, University of Wollongong, North Wollongong, NSW 2500, Australia

.

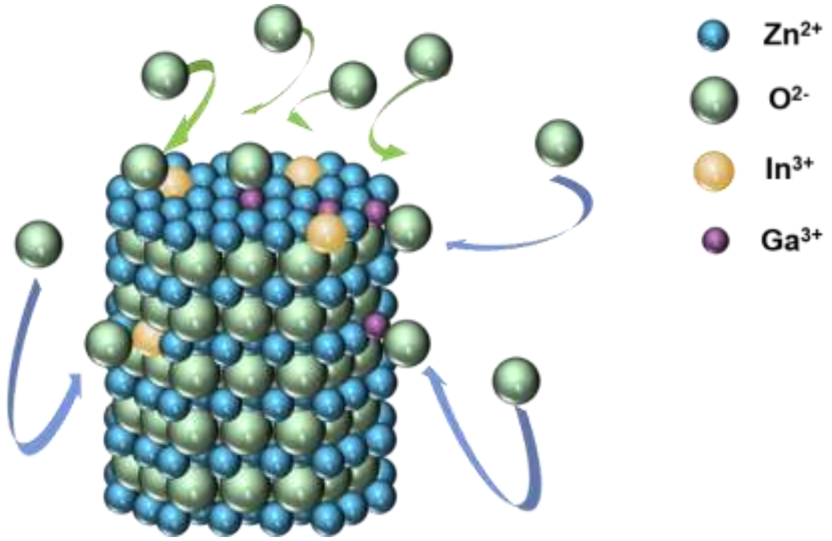

Figure S1. Schematic illustrating the growth of doped ZnO NRs during the hydrothermal process. When the trivalent dopants substitute the divalent Zn sites, oxygen adsorbs more readily on the growing surface. Consequently, the vertical and lateral growth rates of the doped NRs increase.

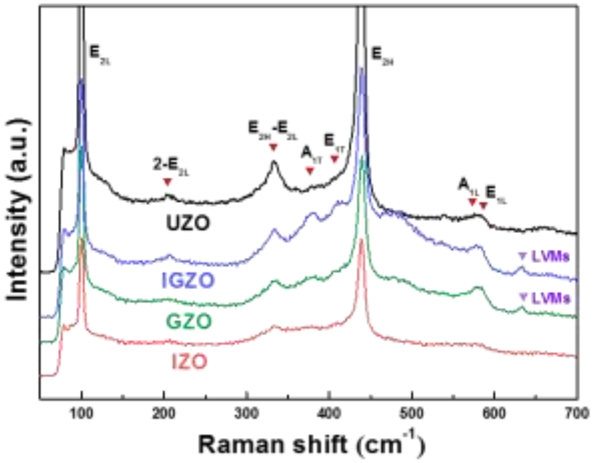

Figure S2. Raman spectra of the UZO, IZO, GZO, and IGZO NRs. The Raman spectra were indexed with all ZnO NRs. For all NRs, the ZnO peaks were located at 100 and 439 cm^{-1}. The latter may be attributed to the low and high E_2 modes (E_{2L} and E_{2H}, respectively) of nonpolar optical phonons, which indicate that ZnO NRs are monocrystalline. For the IZO NRs, all peaks intensities and positions are similar to those of UZO NRs except for the E_{2H}-E_{2L} peak. On the other hand, for the GZO NR, Ga doping introduces three additional peaks related to the Ga content. Especially, the peak located at 629 cm^{-1} may be assigned as local vibrational modes (LVMs) involving Ga point defects such as substitutional Ga (Ga_{Zn}) and/or Ga interstitials (Ga_i).

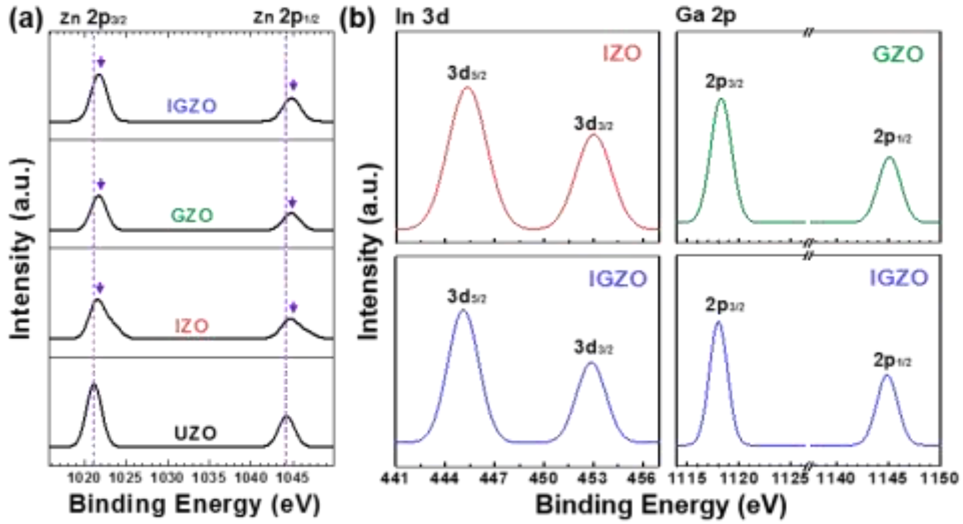

Figure S3. (a) Zn 2p XPS spectra of the UZO, IZO, GZO, and IGZO NRs and (b) In 3d and Ga 2p spectra of the IZO, GZO, and IGZO NRs.

Table S1. XRD peak positions, FWHM of (002) peak, and average c-lattice parameters of the NRs.

Sample	(002) peak position	FWHM	Calculated c-axis parameter
UZO	34.44°	0.273	5.204 nm
IZO	34.42°	0.29	5.207 nm
GZO	34.52°	0.20	5.192 nm
IGZO	34.45°	0.301	5.203 nm

Table S2. XPS peak positions of the OI, OII, and OIII sub-peaks deconvoluted from the O 1s peak, and the relative integrated intensity ratios of the UZO, IZO, GZO, and IGZO NRs.

Sample	O_I		O_{II}		O_{III}	
	Position [eV]	Ratio [O_I/O_{tot}]	Position [eV]	Ratio [O_{II}/O_{tot}]	Position [eV]	Ratio [O_{III}/O_{tot}]
UZO	529.7	0.57	531.3	0.38	532.5	0.05
IZO	530.4	0.42	532.3	0.47	533.7	0.11
GZO	530.2	0.60	531.9	0.33	533.5	0.07
IGZO	530.2	0.61	531.8	0.34	533.2	0.05
